# Unravelling Mechanisms of Oxinflammation Induced by Heavy Metals

**DOI:** 10.3390/metabo16050319

**Published:** 2026-05-09

**Authors:** Luiz Otávio Guimarães-Ervilha, Mírian Quintão Assis, Izabela da Silva Lopes, Thainá Iasbik-Lima, João Victor Leles Faria, Ana Cláudia Ferreira Souza, Mariana Machado-Neves

**Affiliations:** 1 Department of General Biology, Federal University of Viçosa, Viçosa 36570-900, Brazil; mirian.assis@ufv.br (M.Q.A.); izabela.s.lopes@ufv.br (I.d.S.L.); thaina.iasbik@ufv.br (T.I.-L.); joao.leles@ufv.br (J.V.L.F.); 2 Department of Animal Biology, Federal Rural University of Rio de Janeiro, Seropédica 23897-000, Brazil; ana.clfs@gmail.com

**Keywords:** oxidative stress, mitochondrial dysfunction, inflammation, metabolites, aluminum, arsenic, cadmium, lead, mercury, nickel

## Abstract

Exposure to heavy metals remains a significant public health concern due to their environmental persistence, bioaccumulation, and ability to interfere with essential cellular processes. A large part of metal-induced toxicity converges on the establishment of a chronic oxinflammatory state, driven by the reciprocal interaction between oxidative stress and inflammation. In this review, we synthesize current mechanistic evidence describing how toxic metals, including aluminum, arsenic, cadmium, lead, mercury, and nickel, disrupt redox homeostasis, impair cellular integrity, and activate inflammatory signaling pathways. These metals promote the excessive generation of reactive oxygen and nitrogen species through multiple mechanisms, including mitochondrial dysfunction, displacement of essential metal cofactors, and inhibition of antioxidant systems. The resulting molecular damage leads to the formation of damage-associated molecular patterns (DAMPs), which activate redox-sensitive transcription factors and inflammatory cascades. Importantly, emerging metabolomic evidence indicates that these processes are accompanied by coordinated metabolic reprogramming involving amino acid, lipid, and energy metabolism, as well as microbiota-derived metabolites. These metabolic alterations not only reflect cellular adaptation to stress but also actively contribute to the propagation of a systemic inflammatory state. An integrated oxinflammatory and metabolic response underlies structural and functional alterations across multiple organ systems, including the liver, kidneys, cardiovascular system, nervous system, and reproductive organs. Persistent exposure, even at low doses, sustains this often subclinical and chronic process, reinforcing the need to understand metabolic changes as central components of metal-induced toxicity.

## 1. Introduction

Heavy metal contamination poses a challenge to human and animal health. These elements comprise a chemical group that exhibits relatively high density (>5 g cm^−3^) and peculiar physical properties [1]. However, in environmental toxicology, the term heavy metals refers to a group of metallic elements, including metalloids, that are associated with environmental toxicity and persistence and do not strictly correspond to a single physicochemical definition [2]. These toxic metals exhibit high persistence, dispersion, resistance to degradation, and the ability to accumulate in tissues and interfere with metabolic processes, being highly toxic even at low concentrations [3,4,5]. Once in the body, they are not subject to classical metabolic degradation. Instead, their dynamics involve processes of absorption, systemic transport, complexation with reducing molecules, and tissue redistribution [6]. These elements can interact with proteins, lipids, and nucleic acids, as well as displace physiological metal cofactors and disrupt essential catalytic centers [7,8], leading to prolonged exposure resulting in structural and functional damage [9]. In many cases, these alterations do not manifest immediately but evolve into chronic and subclinical processes [10]. In this context, elements such as cadmium (Cd), arsenic (As), lead (Pb), nickel (Ni), and mercury (Hg) are commonly regarded as heavy metals, whereas aluminum (Al), although not strictly classified as a heavy metal, is also of environmental concern due to its potential toxicity under certain conditions. They are widely distributed in water, soil, air, and food sources, both naturally and anthropogenically [7,11].

This interaction between heavy metals and cellular components promotes the generation of reactive oxygen species (ROS) and reactive nitrogen species (RNS) [12,13], with the disruption of redox homeostasis being central to metal toxicity. Oxidative stress generated promotes the formation of secondary bioactive metabolites derived from lipid, protein, and nucleic acid oxidation, which can modify proteins and cellular structures [14]. In parallel, inflammation plays an ambiguous role in heavy metal toxicity. Initially, it acts as a reparative response to cellular damage, but under persistent exposure to metals, it evolves into a chronic inflammatory state [15,16,17]. Apparently, the interdependence between oxidative stress and inflammation establishes a feedback loop that may amplify cellular damage and sustain long-lasting metabolic alterations. Thus, oxinflammation mediated by heavy metals may affect metabolic pathways by eliciting systemic inflammation, which, in turn, disrupts the production of metabolic intermediates [16]. For that reason, metabolomic approaches may provide a framework for identifying and interpreting the mechanisms mediated by metabolites generated by exposure to heavy metals.

Several studies have focused on filling knowledge gaps regarding metal exposure and its relationship with systemic disorders [17,18,19] while also considering patterns of oxidative stress and inflammation [17,20]. This work compiles information from the available literature on the redox and inflammatory responses associated with heavy metal toxicity, as well as the main metabolites involved in those processes. Our main question was as follows: Does exposure to heavy metals initiate damage through oxidative stress or through inflammation? Therefore, this review aimed to determine whether oxidative stress acts as a precursor or a concomitant event to inflammation under metal contamination. Moreover, we characterize the temporal sequence of events and the main altered metabolites resulting from these processes. Recent advances in metabolomics have provided insights into how heavy metal exposure remodels cellular metabolism, enabling the identification of metabolic signatures associated with oxidative stress, inflammation, and metabolic pathways.

## 2. General Mechanisms of Oxidative Stress Induced by Heavy Metals

Contact with heavy metals occurs primarily through ingestion, followed by inhalation and dermal exposure [21,22]. Once inside the cell, heavy metals, such as Al, As, Cd, Hg, Ni and Pb, pass through physiological transporters present in the lipid bilayer, including divalent metal ion transporters 1 (DMT1) [23]; members of the ZIP family (SLC39) (e.g., ZIP8 and ZIP14) [24,25]; calcium (Ca^2+^) channels, such as member 6 of the vanilloid subfamily of transient receptor potential (TRPV) [26]; aquaporins [27]; and neutral amino acid transporters that transport metal-cysteine complexes, including the L1-type amino acid transporter [28].

**Figure 1:**
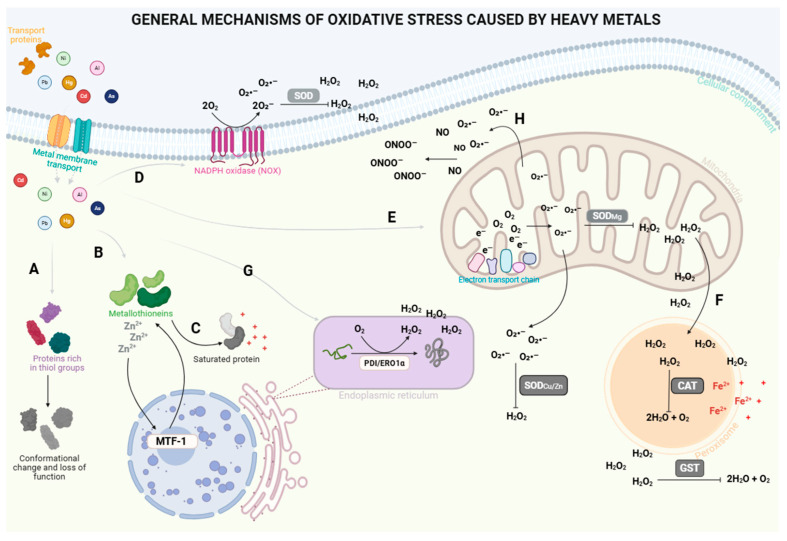
General mechanisms of oxidative stress caused by heavy metals. Aluminum (Al), Arsenic (As), Cadmium (Cd), Lead (Pb), Mercury (Hg), and Nickel (Ni) can enter cells through transporter proteins and membrane transport systems, disrupting redox homeostasis by increasing the generation of reactive oxygen species (ROS) and reactive nitrogen species (RNS) and weakening the antioxidant system. (**A**) In the cell, heavy metals interact with thiol-rich proteins, such as antioxidant enzymes, causing conformational changes and loss of activity. (**B**) Heavy metals also induce or bind to metallothioneins (MTs), proteins that buffer metal ions, compromising their functionality. (**C**) By competing with zinc (Zn^2+^) at binding sites, heavy metals can displace the ion from proteins and/or saturate the binding capacity of metallothionein, increasing the availability of bioactive metals and triggering the activation and nuclear signaling of metal-responsive transcription factor-1 (MTF-1), a transcriptional regulator of MT genes. (**D**) Heavy metals can stimulate ROS sources associated with the plasma membrane, particularly NADPH oxidase (NOX). (**E**) Mitochondria represent a central site of amplification, since metals impair the electron transport chain, increasing electron leakage and ROS generation. (**F**) In parallel, heavy metals influence peroxisomal redox metabolism by disrupting organelle homeostasis. (**G**) Heavy metals can induce oxidative stress in the endoplasmic reticulum by affecting protein folding pathways, generating ROS. (**H**) Heavy metals also exacerbate nitrosative stress by promoting RNS formation. O_2_^•−^: Superoxide anion; H_2_O_2_: Hydrogen peroxide; NO: Nitric oxide; ONOO^-^: Peroxynitrite; SOD: Superoxide dismutase; CAT: Catalase; GST: Glutathione S-transferase; PDI: Protein disulfide isomerase; ERO1α: Endoplasmic reticulum oxidoreductin 1 alpha. Created with BioRender.com.

In the cytosol, heavy metals such as Hg, Pb, and Ni tend to have a strong binding to the thiol/-SH groups of cysteine residues in structural proteins, antioxidant enzymes, and reduced glutathione (GSH), an important intracellular redox buffer (Figure 1A) [29,30,31,32,33]. Due to the high affinity of metal ions for sulfur ligands, stable metal-thiol complexes are formed, leading to structural and functional alterations in several proteins related to antioxidant defense. This interaction may represent the initial event in metal-induced toxicity. In the case of metal-thiol complex formation with GSH, the cellular reducing pool decreases, lowering the GSH/oxidized glutathione (GSSG) ratio and compromising the overall redox potential of the cell [34]. The resulting alterations from this binding inactivate antioxidant enzymes sensitive to the oxidation of thiols or selenothiols, such as glutathione peroxidase (GPx), which has selenocysteine in the active site; glutathione reductase (GR); and glutathione S-transferase (GST). They also disrupt essential metal centers of other redox proteins, such as copper (Cu^2+^)/zinc (Zn^2+^) superoxide dismutase (SOD) [35,36]. In these latter cases, the competitive displacement of Zn^2+^ or Cu^2+^ by Cd^2+^, Pb^2+^, or Hg^2+^ destabilizes the enzyme folding and disrupts the dismutation of the superoxide radical (O_2_^•−^) [37,38]. The heavy metals can also interfere with the homeostasis of other divalent cations, such as Ca^2+^, magnesium (Mg^2+^), and iron (Fe^2+^) [39,40]. By mimicking these ions, heavy metals disrupt Ca^2+^-dependent signaling pathways, inhibit ATPases, and compromise transmembrane transport, cell membrane integrity, and intracellular communication [41].

Although it does not contain reactive thiol groups in its active site, catalase (CAT) is indirectly affected by the oxidation of the heme group, the modification of neighboring cysteine and methionine residues, and the consequent conformational change [3]. The result of this network of interactions is the collapse of enzymatic antioxidant systems, leading to the accumulation of reactive species such as O_2_^•−^ and hydrogen peroxide (H_2_O_2_) [42]. This initial disruption of the redox balance serves as the primary molecular trigger of metal-induced oxidative stress [34].

In parallel, the accumulation of metals in the cytosol increases the intracellular pool of labile Zn^2+^ (Figure 1B) through metal-Zn competition at protein binding sites. This increase in labile Zn^2+^ in the cytosol, along with the increase in other metal ions, is detected by metal-responsive transcription factor-1 (MTF-1), which undergoes Zn-dependent activation and translocates to the nucleus, where it binds to metal-responsive elements (MREs) and induces the transcription of metallothioneins (MTs), such as MT-I and MT-II [43,44]. Initially, MT-I and MT-II act as chelators and antioxidants, binding to metal ions and stabilizing them through highly stable metal-thiol bonds [45]. However, prolonged exposure can saturate and oxidize thiol sites in MTs, promoting the progressive release of previously sequestered metals (Figure 1C) [46]. This release may favor metal-catalyzed redox reactions involving transition metals, contributing to oxidative stress through localized oxidation processes [22], transforming MTs from cytoprotective systems into pro-oxidant reservoirs [47,48]. This self-reinforcing cycle prolongs redox dysfunction and contributes to the chronic nature of metal-induced oxidative stress [46].

At the plasma membrane, ionic imbalances induced by heavy metals activate oxidases [49]. Among these enzymes, NADPH oxidases (NOXs) stand out (Figure 1D), whose physiological function is the controlled generation of O_2_^•−^. Some heavy metals bind directly to the thiol groups of the regulatory subunits of oxidases, deregulating the assembly of NOXs and displacing or oxidizing elements present in these enzymes, such as Zn^2+^ and Fe^2+^, affecting the interaction of the oxidase with its electron donors [50,51]. A typical example of this family is NOX2. Under basal conditions, the catalytic subunits of NOX2 are associated with the membrane, while the regulatory subunits remain in the cytosol. Phosphorylation of the p47phox protein, primarily by the action of protein kinase C (PKC), promotes the translocation of these cytosolic subunits to the plasma membrane [52]. Once assembled, NOX2 catalyzes the transfer of electrons from cytosolic NADPH to molecular oxygen, generating O_2_^•−^ in the perimembrane space, where it is rapidly converted to H_2_O_2_ by spontaneous dismutation or by SOD activity [53]. In the presence of heavy metals, like As and Cd, the activity of NOXs is deregulated. The result is aberrant and/or sustained activation of these oxidases, with increased ROS production [54,55].

Heavy metal toxicity is also associated with organelle damage, particularly its effects on mitochondria (Figure 1E). These elements can reach the organelle via the mitochondrial Ca^2+^ uniporter, divalent cation transporters such as manganese (Mn^2+^), or through intracellular import. They may cross the mitochondrial membrane bound to glutathione-metal and MT-metal complexes [56,57]. In mitochondria, Al, As, Cd, Hg, Ni and Pb can interfere with the organization and functioning of the electron transport chain (ETC), creating conditions for electron leakage and exacerbated ROS generation (Figure 1E) [58,59,60]. Two sites of the ETC are particularly sensitive: Complex I (Reduced nicotinamide adenine dinucleotide (NADH): ubiquinone oxidoreductase) and Complex III (cytochrome bc_1_ complex). Leakage in Complex I increases due to reverse electron transport (RET) and an increase in mitochondrial membrane potential (ΔΨm), where succinate, a substrate for the reactions of Complex II, increases the ubiquinol/ubiquinone ratio (QH_2_/Q) [61]. Consequently, in Complex III, an increase in the QH_2_/Q or the limitation of electron flow induces the accumulation of stabilized semiquinone at the Qₒ site in the intermembrane space [62]. This process results in the univalent transfer of O_2_^•−^ into the intermembrane space [62]. Thus, by disrupting redox centers, increasing ΔΨm, favoring RET, and compromising mitochondrial antioxidant systems, heavy metals can convert mitochondria into a central and amplified source of metal-induced oxidative stress. Thus, the ROS crosstalk between mitochondria and NOXs can amplify signaling, propagate oxidative damage, and sensitize inflammatory pathways [54].

Unlike mitochondria, which generate O_2_^•−^ by leakage, the peroxisome produces H_2_O_2_ directly as a byproduct of oxidases. Physiologically, within the peroxisome, the first step of peroxisomal β-oxidation generates H_2_O_2_ [63]. Under basal conditions, peroxisomal CAT converts H_2_O_2_ to H_2_O + ½ O_2_. However, in the presence of heavy metals, CAT is inhibited by the displacement or oxidation of Fe^3+^ from the heme group and by the oxidation of thiols, increasing intraperoxisomal H_2_O_2_ and, consequently, its leakage (Figure 1F) [64]. Since metals also promote peroxisomal proliferation by activating the peroxisome proliferator-activated receptor alpha (PPARα) transcription factor [49] and inducing other oxidases, the H_2_O_2_ flux increases and the CAT capacity decreases, losing its ability to remove H_2_O_2_ from the environment [49]. The gradient pushes H_2_O_2_ into the cytosol, where it triggers the release of additional ROS by complexes I and III of the ETC, a process known as ROS-induced ROS release (RIRR) [65].

The endoplasmic reticulum (ER) is another natural source of ROS and a potential target for heavy metals such as Cd and Ni (Figure 1G). Oxidoreductases form disulfide bridges in newly synthesized proteins during folding, with protein disulfide isomerase (PDI) being the best-known [66]. The catalytic activity of PDI depends on the action of proteins from the ER oxidoreductin 1 (ERO1) family, represented by the ERO1α and ERO1β isoforms. These enzymes exhibit high oxidative capacity due to the presence of the cofactor flavin adenine dinucleotide (FAD), which transfers electrons from the cysteine residues of PDI to O_2_, the final electron acceptor [67]. Thus, H_2_O_2_ is generated in the ER lumen and is naturally neutralized by antioxidant enzymes [68]. The presence of heavy metals compromises this balance. Metals can bind to PDI’s cysteine residues, suppressing its activity and leading to the accumulation of misfolded proteins in the reticulum [68]. This dysfunction triggers the unfolded protein response (UPR). Persistent UPR activation can lead to ERO1α overexpression, thus intensifying H_2_O_2_ production [69]. Changes in the cellular redox state, resulting from excessive H_2_O_2_ production, can also modulate the activity of Ca^2+^ transporter proteins, culminating in the opening of inositol triphosphate receptor (IP_3_R) channels located in the ER membrane, as well as an increase in the activity of the mitochondrial Ca^2+^ uniporter, responsible for the selective entry of Ca^2+^ into the mitochondrial matrix. Thus, Ca^2+^ released by the IP_3_Rs is taken up in the mitochondria and intensifies the generation of ROS, which, in turn, amplifies mitochondrial Ca^2+^ uniporter activity, establishing a feedback loop [70]. Therefore, the ER and mitochondria form a redox communication circuit. H_2_O_2_ generated in the ER causes mitochondrial damage and high production of ROS [71,72].

The generation of RNS activates redox-sensitive inflammatory pathways, promoting the induction of inducible nitric oxide synthase (iNOS), increasing nitric oxide (NO) production. Under these conditions, O_2_^•−^, present in excess in the cytosol due to the interaction of heavy metals, reacts with NO, directly competing with its dismutation, forming peroxynitrite (ONOO^−^; Figure 1H) [73]. Among its main targets are enzymes containing iron-sulfur centers, whose inactivation occurs through the oxidation of iron and the rupture of bonds with inorganic sulfur [74]. ONOO^−^ promotes the nitration of tyrosine residues in proteins, forming 3-nitrotyrosine, which not only reflects protein damage but also alters the catalytic and structural properties of mitochondrial and cytosolic enzymes, contributing to metabolic dysfunction and increased production of reactive species [73].

Thus, the initial damage caused by exposure to heavy metals appears to arise from interactions between the metals and the antioxidant system, leading to the formation of reactive species. The interaction of these reactive species with various organelles and cellular structures can generate secondary metabolites that exacerbate cellular damage and danger signals, thereby activating inflammatory pathways [75,76,77]. The following section reviews the main metabolites generated by ROS and RNS that contribute to inflammation and how this process occurs, fostering the oxinflammatory cycle.

## 3. Oxidative Metabolites Generated by a Pro-Oxidant Environment Mediated by Heavy Metals

ROS and RNS generated by heavy metal interactions are highly unstable and have limited spatial range, reacting preferentially at their site of origin [78,79]. These reactions lead to the formation of secondary oxidative metabolites that propagate cellular damage. Thus, the resulting cellular damage disrupts the functional integrity of organelles, leading to cellular injury and initiating an inflammatory repair process [17].

In membranes, metal-catalyzed oxidative reactions promote lipid peroxidation, especially in regions rich in polyunsaturated fatty acids (PUFA), compromising membrane asymmetrical organization, integrity, and permeability [80,81]. As a result, membranes become functionally unstable, leading to ionic imbalance, compromised vesicular transport, and intracellular compartmentalization. For example, in mitochondria, changes in membrane permeability increase ROS leakage [82], while in lysosomes, increased permeability of the organelle allows the escape of redox-active metal ions, such as Fe^2+^, and acid hydrolases into the cytosol [83].

Regarding proteins, reactive species promote post-translational modifications that affect structural stability and function. Cysteine, histidine, and lysine residues are preferential targets, undergoing progressive oxidation, formation of aberrant disulfide bridges, or covalent adduction by reactive aldehydes generated as secondary metabolites of lipid peroxidation [84,85]. In metabolic enzymes and components of the respiratory chain, the reversibility of these alterations is often limited, leading to the accumulation of dysfunctional proteins that exceed the capacity of chaperone systems and proteolytic degradation [85,86]. In DNA, reactive species interact with nitrogenous bases and the phosphodiester backbone, leading to modifications of purine and pyrimidine bases, single-strand breaks, and cross-linking [87]. These lesions compromise the integrity of genetic material, activate repair mechanisms, and promote genomic instability [88]. Mitochondrial DNA is particularly susceptible to oxidative damage due to its proximity to sources of reactive species and its lack of protection systems equivalent to those in the nucleus [89].

These events lead to disruption of cellular homeostasis and the accumulation of oxidative metabolites [90]. The oxidation of PUFA leads to the formation of reactive aldehydes, such as malondialdehyde (MDA) and 4-hydroxynonenal (4-HNE), which covalently bind to nucleophilic protein residues, altering their conformation and function [91]. Simultaneously, proteins undergo carbonylation and nitration, including the formation of nitrotyrosine via ONOO^−^, while nuclear and mitochondrial DNA accumulates oxidative lesions, such as 8-oxo-2′-deoxyguanosine (8-Oxo-dG) [92]. This state creates the conditions for the imminent release of intracellular danger signals. The release of oxidized mitochondrial components, the exposure of intracellular molecules, and the alteration of membrane integrity convert oxidative damage into a trigger for danger recognition pathways. Thus, ROS- and RNS-induced damage acts as a link between initial oxidative stress and the subsequent activation of the reparative inflammatory response [17,20]. Also, the oxidation of PUFAs can generate highly reactive γ-ketoaldehydes known as isolevuglandins (IsoLGs) [93]. These compounds arise from the rearrangement of lipid endoperoxides formed during oxidative stress and are among the most reactive products of lipid peroxidation. IsoLGs react rapidly with lysine residues in proteins, forming stable adducts that can alter protein structure and function. These adducts are immunogenic and have been shown to promote inflammatory responses [94]. The accumulation of IsoLG-modified proteins has been associated with chronic inflammatory states and implicated in the pathogenesis of hypertension and cardiovascular disease [95]. Although IsoLGs are increasingly recognized as important mediators linking lipid peroxidation to immune activation and inflammation, their potential involvement in heavy metal-induced oxidative damage remains unexplored.

To minimize the damage caused by reactive species, the cell activates adaptive mechanisms, mainly mediated by nuclear erythroid factor 2-related factor 2 (Nrf2) [96]. Under basal conditions, Nrf2 remains in the cytosol, associated with the repressor protein Keap1, and is continuously directed to proteasomal degradation [97]. Apparently, exposure to heavy metals induces modifications in the cysteine residues of Keap1, leading to dissociation of the Keap1-Nrf2 complex and allowing the nuclear translocation of Nrf2 [98]. Once in the nucleus, Nrf2 induces the transcription of cytoprotective genes involved in glutathione synthesis, xenobiotic metabolism, and metal detoxification.

Although Nrf2 activation is a compensatory mechanism to restore redox homeostasis, chronic or intense exposure to metals can compromise Nrf2 signaling in the nucleus. This impairment results from the progressive depletion of reducing cofactors, persistent oxidation of regulatory proteins, epigenetic alterations, and interference from dominant inflammatory pathways, such as nuclear factor-κB (NF-κB), which compete for transcriptional coactivators and/or repress the transcription of antioxidant genes [96,99]. Consequently, the nuclear response mediated by Nrf2 becomes insufficient to neutralize the oxidative load resulting from chronic metal exposure, favoring a transition from an adaptive state to a failure state [99].

## 4. Inflammatory Mechanisms Mediated by Metabolites Generated by Heavy Metal-Induced Oxidative Stress

**Figure 2:**
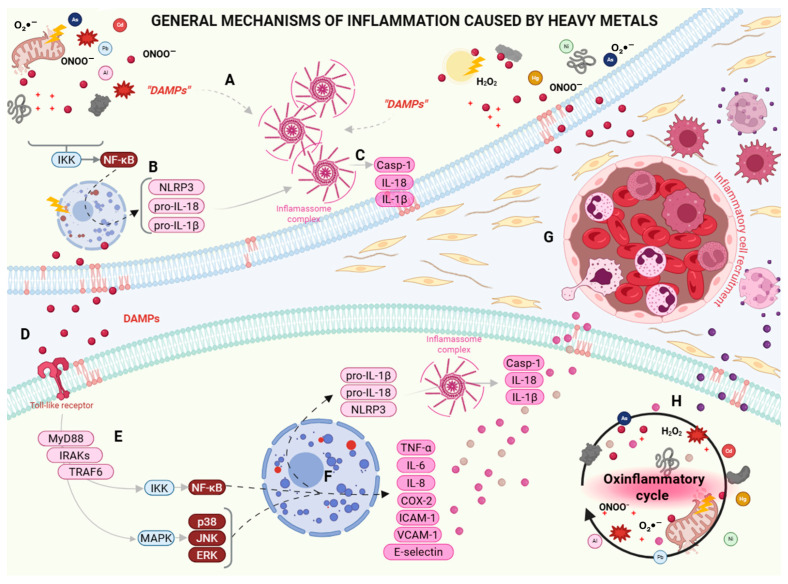
General mechanisms of heavy metal-induced inflammation. Aluminum (Al), arsenic (As), cadmium (Cd), lead (Pb), mercury (Hg), and nickel (Ni) promote inflammatory responses by coupling oxidative stress, cellular damage, and inflammatory signaling pathways. (**A**) Heavy metal-induced oxidative and nitrosative stress leads to cellular damage and the release of damage-associated molecular patterns (DAMPs), which act as danger signals. (**B**) DAMPs activate sensitive pathways, including IKK/NF-κB, inducing the transcription of inflammasome components such as NLRP3, as well as inactive cytokine precursors, pro-IL-1β and pro-IL-18. (**C**) DAMPs and intracellular stress signals promote the assembly of the NLRP3 inflammasome complex, leading to the activation of caspase-1 (Casp-1) and the subsequent maturation and release of IL-1β and IL-18. (**D**) Extracellular DAMPs are recognized by Toll-like receptors (TLRs) in the plasma membrane. (**E**) TLRs activate the MyD88 pathway, involving IL-1 receptor-associated kinases (IRAKs) and TNF receptor-associated factor 6 (TRAF6), which converge on the IKK/NF-κB and MAPK (mitogen-activated protein kinase) cascades, including p38, JNK, and ERK. (**F**) Nuclear translocation of NF-κB and MAPK-regulated transcription factors drives the expression of pro-inflammatory mediators, including TNF-α, IL-6, IL-8, COX-2, adhesion molecules (ICAM-1 and VCAM-1), and E-selectin, promoting leukocyte activation and recruitment. Parallel activation of the inflammasome pathway results in increased release of Casp-1-dependent cytokines, amplifying local and systemic inflammation. (**G**) Inflammatory mediators promote endothelial activation, leading to increased expression of adhesion molecules and facilitating leukocyte adhesion and recruitment to the affected tissue. (**H**) Persistent exposure to metals sustains a self-perpetuating oxinflammatory cycle, in which the generation of reactive oxygen species (ROS) and reactive nitrogen species (RNS) reinforces inflammatory signaling, mitochondrial dysfunction, and further release of DAMPs, ultimately contributing to chronic inflammation and tissue injury. COX-2: cyclooxygenase-2; ERK: extracellular signal-regulated kinase; H_2_O_2_, hydrogen peroxide; ICAM-1: intercellular adhesion molecule 1; IKK: IκB kinase; IL-18: interleukin-18; IL-1β: interleukin-1 beta; IL-6: interleukin-6; IL-8: interleukin-8; JNK: c-Jun N-terminal kinase; MAPK: mitogen-activated protein kinase; MyD88: myeloid differentiation primary response 88; NF-κB: nuclear factor-κB; NLRP3: NOD-like receptor family pyrin domain-containing 3; O_2_^•−^, superoxide anion; ONOO^-^, peroxynitrite; p38: p38 mitogen-activated protein kinase; TNF-α: tumor necrosis factor alpha; VCAM-1: vascular cell adhesion molecule 1. Created with BioRender.com.

Although heavy metals can directly modify proteins, most structural damage is generated through the formation of secondary oxidative metabolites. These danger signals activate inflammatory pathways, converting chemical damage into immunological language through damage-associated molecular patterns (DAMPs; Figure 2A), pattern recognition receptors (PRRs), and cytosolic platforms such as inflammasomes [100,101]. These events trigger an intracellular response that transforms oxidative damage into inflammasome activation. By compromising cell integrity, extracellular DAMPs activate Toll-like receptors (TLRs), propagate inflammation in the tissue/organ, recruit immune cells, and consolidate an oxinflammatory cycle [102]. Thus, metal-induced stress is initially a biochemical disturbance. Its pathological impact stems from the conversion of redox imbalance into structural damage, affecting lipids, proteins, nucleic acids, and organelles, thereby generating DAMPs and activating inflammatory pathways [17,20].

Before any inflammasome can be activated, its components must be available. The main inflammasome involved in this response is NLRP3, which comprises three components: the NLRP3 sensor, the ASC adapter, and pro-caspase-1 [101]. Inflammasome availability depends on transcriptional activation, mainly controlled by the transcription factor NF-Κb (Figure 2B) [103,104]. In cells exposed to heavy metals, the products of ROS- and RNS-mediated molecular damage act as signals that activate redox-sensitive pathways [55,105,106,107,108], converging on the IκB kinase complex (IKK). Activation of the IKK complex promotes phosphorylation and subsequent degradation of the inhibitor of NF-κB (IκBα), releasing NF-κB for nuclear translocation [109]. In the nucleus, NF-κB induces the expression of structural components of the NLRP3 inflammasome and precursor forms of pro-inflammatory cytokines, such as interleukin-1β (pro-IL-1β) and interleukin-18 (pro-IL-18), establishing an initial inflammatory state [110].

Once NLRP3 and pro-inflammatory cytokines are available, the cell becomes sensitive to intracellular signals that reflect structural and metabolic failures (Figure 2C). NLRP3 acts as a cellular stress sensor, responding to multiple alterations [111]. The main signals of inflammasome activation include increased mtROS and the release of oxidized mtDNA into the cytosol, lipid oxidation, and ionic imbalance [103,112]. These events promote conformational changes in NLRP3 that favor its oligomerization and the recruitment of the ASC adapter, culminating in the formation of the functional inflammasome complex [113]. Inflammasome assembly activates effector caspase-1, which cleaves pro-IL-1β and pro-IL-18 into their active forms, IL-1β and IL-18. In addition, caspase-1 cleaves gasdermin D, forming plasma membrane pores that can induce pyroptosis in the damaged cell [113].

DAMPs cease to be exclusively intracellular and reach extracellular spaces and tissue (Figure 2D). This communication occurs through the release of intracellular molecules from the damaged cell to the extracellular domain via two main mechanisms: passive release via cell death or active secretion by viable cells under stress. In the initial subclinical effects of heavy metals on the redox system, even in still viable cells, there is active secretion of DAMPs, either through extracellular vesicles or through membrane channels and transporters [114]. DAMPs function as molecular messengers of oxidative damage. They can be recognized by PRRs expressed on neighboring cells and on cells of the innate immune system [113], such as TLRs, type I transmembrane glycoproteins widely distributed in tissues, and other receptors [115].

TLRs play a central role in inflammatory processes. In particular, TLR2 and TLR4 can recognize a wide range of DAMPs derived from oxidative damage [116]. Although no conclusive studies exist, it is believed that these receptors do not recognize the metal ions themselves, but rather the molecular patterns generated by the damage they cause. The recognition of DAMPs by TLRs amplifies inflammation [115]. Resident macrophages, endothelial cells, and adjacent epithelial cells express TLRs and respond to damage occurring in neighboring cells [117], even if they have not directly absorbed the metals. Thus, inflammation becomes diffuse, consistent with the patterns of chronic inflammation observed in heavy metal poisoning. Nevertheless, the activation of TLRs by extracellular DAMPs establishes a link between initial oxidative damage and the activation of complex inflammatory programs, leading to chronic inflammation and tissue remodeling [118].

The recognition of DAMPs by TLRs is amplified through signaling pathways such as NF-κB and mitogen-activated protein kinases (MAPKs; Figure 2E) [100,115]. Activated TLRs recruit cytosolic adaptor proteins that function as signaling platforms, with the myeloid differentiation primary response 88 (MyD88)-dependent pathway apparently being the most relevant in the context of heavy metal-induced inflammation [119,120,121]. This interaction initiates the assembly of the so-called “mydosome,” a multiprotein complex that includes kinases from the IL-1 receptor-associated kinases (IRAK) family, mainly IRAK4 and IRAK1 [122]. The sequential activation of these kinases promotes the recruitment of tumor necrosis factor receptor-associated factor 6 (TRAF6), which propagates the inflammatory signal [122,123]. This, in turn, leads to the recruitment of additional kinases. Among these, the TAK1-TABs complex stands out, functioning as a bifurcation point in signaling [123]. In TAK1, two main pathways are activated in a coordinated manner: the NF-κB and MAPK pathways [122]. Following NF-κB activation, TAK1 phosphorylates the IKK complex. Consequently, NF-κB, previously retained in the cytosol, is released and translocated to the nucleus, where it acts as a transcription factor [109,115,122]. In the context of heavy metal exposure, this axis is particularly relevant because it provides the activation signal necessary for the sustained expression of pro-inflammatory cytokines and components of the NLRP3 inflammasome. In parallel, in the MAPK pathway, the activation of p38, JNK, and ERK kinases occurs, which act in the nucleus, phosphorylating transcription factors such as activator protein 1 (AP-1) (c-Jun/c-Fos), activating transcription factor 2 (ATF-2), and Elk-1 [122,124]. While NF-κB acts directly as a transcription factor, MAPKs regulate both transcription and mRNA stability [115]. Thus, the simultaneous activation of NF-κB and MAPKs confers a persistent character to the inflammatory response induced by heavy metals.

Activation of NF-κB and MAPK pathways culminates in the transcriptional reprogramming of resident and immune cells, resulting in the production of cytokines, chemokines, and inflammatory mediators (Figure 2F). This set represents the main mechanism by which oxidative damage, induced by heavy metals, is converted into an inflammatory response [125]. Among the cytokines, tumor necrosis factor-alpha (TNF-α) and interleukin-6 (IL-6) stand out. TNF-α acts as a central mediator of inflammation, promoting the secondary production of reactive species, vascular dysfunction, and sensitization of other inflammatory pathways [126]. IL-6, in turn, plays a pleiotropic role, integrating local and systemic responses, including metabolic alterations and modulation of immune cell differentiation [127]. In parallel with cytokines, MAPK activation promotes the expression of chemokines. Chemokines from the CXC and CC families establish chemotactic gradients that direct the recruitment of neutrophils, monocytes, and lymphocytes to metal-damaged tissue [128]. This recruitment amplifies the inflammatory response but also introduces new sources of ROS and RNS, intensifying oxidative damage. Activation of the vascular endothelium, which transforms the vascular system into an active participant in inflammation, is crucial. Under the influence of TNF-α and IL-6, endothelial cells increase the expression of adhesion molecules, including intercellular adhesion molecule 1 (ICAM-1), vascular cell adhesion molecule 1 (VCAM-1), and E-selectin, facilitating the migration of inflammatory cells into the tissue [129]. In addition to cytokines and chemokines, inflammatory enzymes such as cyclooxygenase-2 (COX-2) and iNOS are expressed, broadening the spectrum of active mediators in the tissue. COX-2 promotes prostaglandin synthesis, contributing to vasodilation, while iNOS generates large amounts of NO [130]. In an environment already contaminated with heavy metals, NO reacts rapidly with O_2_^•−^ to form the aforementioned ONOO^−^, intensifying oxidative and nitrosative damage to proteins, lipids, and DNA [131]. In this way, the products of the inflammatory response themselves begin to feed back into the oxidative stress induced by the metals.

The presence of pro-inflammatory cytokines in the tissue microenvironment promotes intense recruitment and activation of immune system cells (Figure 2G) [132]. Once activated, these cells begin to produce large amounts of ROS and RNS through their own mechanisms, including the activation of NOXs (especially NOX2), myeloperoxidase activity in neutrophils, and iNOS induction in macrophages [133]. These secondary sources of reactive species, in addition to those generated by the direct effects of metals, drastically increase the tissue’s oxidative load. The sustained increase in ROS and RNS intensifies damage to macromolecules and organelles, perpetuating the formation of intracellular and extracellular DAMPs [134]. Lipids continue to undergo peroxidation, proteins accumulate oxidative and nitrative modifications, and damaged mitochondria remain persistent sources of ROS and oxidized mtDNA. These signals reinforce TLR activation in cells and promote recurrent activation of the NLRP3 inflammasome [135].

The inflammatory response ceases to be an adaptive mechanism to contain damage and becomes a continuous amplifier of tissue injury, establishing the so-called oxinflammation cycle (Figure 2H). Thus, persistent inflammation activation generates oxidative stress, and oxidative stress, in turn, sustains inflammation [136]. In parallel, chronic inflammation induced by heavy metals causes permanent organ damage [137] by significantly interfering with inflammatory resolution mechanisms. Persistent activation of NF-κB and MAPKs maintains the expression of pro-inflammatory cytokines and mediators. At the same time, the effectiveness of cytoprotective and anti-inflammatory pathways, such as those mediated by Nrf2, tends to be progressively reduced due to depletion of cellular antioxidants, inhibition of redox-sensitive enzymes, and continuous exposure to chemical insults. This imbalance favors the transition from potentially reversible inflammation to a state of chronic inflammation with low resolution capacity [136,138]. Over time, the persistence of the oxinflammation cycle promotes structural changes in the tissue. The continuous release of cytokines promotes fibroblast activation and extracellular matrix deposition, contributing to fibrosis and functional loss of the tissue/organ [138]. In metabolically active organs or those with a high capacity for metal bioaccumulation, such as the liver, this process is associated with progressive loss of function, greater susceptibility to other aggressions, and a higher risk of chronic diseases [137]. The maintenance of an oxinflammatory microenvironment creates favorable conditions for persistent mitochondrial dysfunction [139] and genomic instability with epigenetic alterations [140]. Therefore, oxinflammation should not be interpreted merely as a local phenomenon but as a pathological state capable of impacting multiple organ systems over time.

Notably, recent studies reveal that alterations in the metabolism of various pathways, including amino acids, lipids, and energy, induced by exposure to heavy metals can contribute to sustained oxinflammation [75,76,77]. Thus, we also review metabolomics studies that assess the impact of heavy metal exposure on various metabolic pathways and on the generation of metabolites associated with oxinflammation.

## 5. Altered Metabolites and Metabolic Signatures Following Heavy Metal Contamination

Metabolomics is a tool that has been gaining ground in environmental toxicology studies, enabling the comprehensive characterization of biochemical disturbances and the identification of early, sensitive biomarkers of exposure to toxic substances [141,142,143,144]. Metabolomic studies have revealed that exposure to heavy metals induces consistent alterations across key metabolic pathways, including amino acid, lipid, and energy metabolism, as well as microbiota-derived metabolites. Nevertheless, such studies did not explore the data with a focus on redox and inflammatory metabolites. Thus, we reviewed these studies to examine their data and identify the intermediate metabolites associated with the oxinflammation cycle following heavy metal exposure. The studies included in this section were selected through a non-systematic literature search, prioritizing relevant articles that investigate heavy metal exposure and metabolomic alterations, particularly in murine experimental models (Table 1). These changes reflect an integrated cellular response to oxidative stress and inflammation, generating measurable metabolic signatures.

Heavy metal contamination alters amino acid metabolism closely linked to antioxidant defenses, reflecting adaptive responses to redox imbalance [76,151,153,162]. Metabolomic analyses have consistently reported disruptions in pathways involving phenylalanine, tyrosine, and tryptophan following exposure to Al, Cd, and Ni [146,153,159]. These amino acids are key metabolic precursors involved in neurotransmitter synthesis, immune modulation, and redox homeostasis, and their dysregulation contributes to the propagation of oxidative and inflammatory processes.

Lipid metabolism represents another central axis affected by metal exposure. Alterations in glycerophospholipid metabolism, fatty acid β-oxidation, and steroid hormone biosynthesis have been widely reported in response to As, Cd, Ni, and Hg exposure [77,147,152,163]. In a murine model of As-induced toxicity, integrated metabolomic and transcriptomic analyses revealed disruptions in lipid metabolic pathways associated with hepatic triglyceride accumulation and impaired mitochondrial β-oxidation, contributing to liver injury [163]. Similarly, exposure to Al and Cd leads to lipid metabolic remodeling [147,152], including disturbances in bile acid metabolism mediated by alterations in gut microbiota composition and gut–liver axis signaling [77]. Consistently, exposure to Pb and Hg also results in lipid metabolism disruption as a central component of the systemic response to metal toxicity [156,164].

Energy metabolism is also markedly affected. Exposure to As, Hg, and Cd disrupts glycolysis, Krebs cycle intermediates, and pyruvate metabolism [149,151,157]. These alterations are closely associated with mitochondrial dysfunction, a primary target of redox imbalance, which promotes electron leakage from the electron transport chain and amplifies reactive species production.

Recent metabolomic studies further highlight the role of the gut microbiota as a mediator of metal-induced toxicity. Exposure to As induces significant shifts in microbial composition and associated metabolites, including those involved in amino acid biosynthesis (e.g., tyrosine and tryptophan) and lipid metabolism, such as glycerophospholipids and linoleic acid [150]. Integrated microbiome–metabolomics approaches have demonstrated similar disruptions in microbiota–metabolite interactions following exposure to Cd, Pb, Al, and Ni. These changes are associated with alterations in lipid metabolites, amino acids, and bile acids, supporting the role of the gut microbiota as a key interface linking environmental exposure to systemic metabolic dysfunction and inflammation [77,154,155,160,161].

Metabolomics also enables the identification of sex-dependent responses to environmental toxicants [145], providing insights into mechanisms underlying reproductive dysfunction. For example, exposure to As alters metabolites associated with spermatogenesis and steroidogenesis in the testes [75], changes that are closely linked to oxidative stress and inflammatory signaling and may ultimately compromise reproductive function. However, metabolomic data addressing reproductive outcomes associated with other metals remain limited.

It is important to highlight that a detailed analysis of the metabolomic studies included in Table 1 reveals that several altered metabolites not only indicate general metabolic dysfunction but are also associated with oxidative stress and inflammatory pathways, confirming the concept of oxinflammation as an integrated process. Although little explored, alterations in metabolites related to the redox system have been reported for different metals. For example, exposure to Al and As has been associated with alterations in glutathione metabolism as well as in hypoxanthine, xanthine, taurine, and nicotinamide levels, reflecting dysfunction of antioxidant defenses and increased production of reactive species [146,149]. Similarly, exposure to Cd and Hg alters the GSH/GSSG balance and amino acid metabolism, including the arginine and proline pathways, which are linked to redox homeostasis and cellular responses (e.g., cell signaling and immune response) [151,152,157]. In parallel, amino acids such as phenylalanine, tyrosine, histidine, and methionine show consistent dysregulation in models exposed to As and Cd, suggesting disturbances in immune modulation, neurotransmitter synthesis, and adaptation to oxidative stress [75,153]. Lipid metabolism is also part of oxinflammatory signaling, as evidenced by alterations in arachidonic acid, linoleic acid, lysophospholipids, and bile acids in response to exposure to As, Pb, and Ni [150,154,159,160]. These metabolites are involved in membrane remodeling and the generation of lipid mediators that regulate inflammatory pathways. Inflammatory mediators such as prostaglandins and resolvins have been identified in animal models exposed to As and Pb [76,156]. Intermediates of purine metabolism, including uric acid, are altered after exposure to Cd, Hg, and Pb, reflecting both oxidative stress and nucleotide turnover associated with inflammation [151,156,158]. Alterations in energy metabolism further corroborate this integrated response, with metabolites such as acetylcarnitine and phosphoenolpyruvate being affected in experimental models exposed to As and Pb, indicating mitochondrial dysfunction and metabolic adaptation to oxidative stress [75,156].

Collectively, these findings indicate that metabolomic alterations reflect a coordinated process of metabolic reprogramming driven by oxidative stress and inflammatory activation [148,156]. Notably, metabolomic profiles across different metals and biological systems reveal a high degree of convergence in affected pathways. Despite differences in chemical properties and toxicokinetics, metals such as, Cd, Ni, and Hg consistently disrupt metabolic networks involving amino acids, lipids, mitochondrial function, and microbiota-derived metabolites [149,161,164]. This convergence supports the concept that metal-induced toxicity may be characterized by a shared metabolic phenotype associated with oxinflammation. In this context, metabolomic signatures represent not only biomarkers of exposure but also mechanistic indicators of systemic dysfunction. These findings support the concept that metabolic reprogramming is not merely a consequence of metal-induced toxicity but a central component of the oxinflammatory process, actively shaping cellular responses and disease progression. Future studies integrating metabolomics with other omics approaches, including transcriptomics, proteomics, and microbiome analyses, may further elucidate the complex networks linking metal exposure to metabolic dysregulation and disease development.

## 6. Consequences of Heavy Metal-Induced Oxinflammation in Biological Systems

Although the mechanisms of oxidative stress, metabolite formation, and the resulting inflammatory profile are shared across different heavy metals, their biological effects manifest according to each metal’s toxicological properties, including its chemical form and distribution. We synthesize histopathological findings across organs and systems in murine models susceptible to heavy-metal action, along with the resulting oxinflammatory profile.

### 6.1. Aluminum

Al is one of the most abundant elements in the Earth’s crust and is widely used in modern society, from food packaging to pharmaceutical formulations [165]. Although it has no biological function, its systemic bioavailability has increased due to anthropogenic activities, with ingestion of contaminated water and food and inhalation being the main routes of exposure [166]. After absorption, Al^3+^ is transported in the plasma, mainly bound to transferrin, competing with Fe^3+^ for binding sites, thereby interfering with iron homeostasis and favoring its tissue redistribution [167,168].

At a systemic level, Al-induced oxinflammation affects multiple organs through convergent mechanisms. In the liver, exposure to the metal is associated with mitochondrial dysfunction, increased ROS production, lipid peroxidation, and chronic inflammation, culminating in alterations in lipid metabolism and hepatocellular injury [169]. In the male reproductive system, aluminum increases oxidative stress by elevating ROS production and local inflammation, leading to increased levels of pro-inflammatory enzymes such as TNF-α and IL-6. It is related to reduced testosterone levels, reduced steroidogenesis, and impaired spermatogenesis, leading to decreased sperm count, motility, and structural integrity [170]. In the female reproductive system, Al exposure is associated with hormonal imbalance, oxidative stress, and ovarian cell apoptosis, thus compromising oocyte quality and gonadal homeostasis [171,172]. In the cardiovascular system, increased ROS and lipid peroxidation contribute to endothelial dysfunction, inflammatory activation, and vascular remodeling, favoring functional alterations in the heart [173]. In the nervous system, Al can cross the blood–brain barrier, and its accumulation is associated with neurodegenerative diseases, such as Alzheimer’s, and can induce a neuroinflammatory response [174]. In the kidneys, Al exposure promotes tubular and glomerular dysfunction, oxidative damage, impairment of antioxidant defenses, and inflammatory activation, resulting in nephrotoxicity [175]. Taken together, these findings consolidate Al as a systemic inducer of an integrated oxinflammatory response, sustained by redox imbalance, mitochondrial dysfunction, inflammatory signaling pathways such as NF-κB, and metabolic dysregulation in multiple organs, with evidence of pyroptosis.

### 6.2. Arsenic

As is a metalloid that occurs in multiple chemical forms whose reactivity and toxicity are strongly dictated by oxidation state and speciation. In biological and environmental contexts, the most relevant oxidation states are As^3+^ (arsenite; trivalent) and As^5+^ (arsenate; pentavalent), while organic arsenicals (e.g., methylated metabolites) arise from mammalian biotransformation pathways and may either reduce or amplify toxicity depending on their redox state [176,177]. In general, trivalent arsenicals are more thiol-reactive than pentavalent species, enabling stronger interactions with protein cysteine residues and catalytic cofactors, an important feature underpinning both metabolic dysfunction and redox imbalance [176,178].

The liver is a principal site for arsenic biotransformation and, therefore, a major target for combined metabolic and inflammatory injury. The mtROS–NLRP3 pathway described above provides a direct mechanistic link between arsenic exposure and hepatic insulin resistance and inflammatory amplification [179]. The kidney, in turn, is highly vulnerable due to its role in xenobiotic handling and the susceptibility of proximal tubular cells to mitochondrial dysfunction and inflammation. Recent work demonstrated that arsenic exposure induced acute kidney injury, associated with oxidative stress and inflammation, implicating regulatory nodes such as sirtuin 1 (SIRT1) and PINK1/Parkin-dependent mitophagy in modulating injury severity [180]. Cardiovascular toxicity is increasingly understood through the lens of endothelial redox biology. A focused synthesis emphasized that arsenic exposure predisposes to vascular disease states, in part through oxidative stress mechanisms involving NOXs (e.g., NOX2) and endothelial dysfunction, which fit well within oxinflammation as a vascular process [181]. In combination with evidence for arsenic-linked NF-κB activation in vascular cells [182], arsenic can be considered a driver of sustained vascular inflammatory tone, with downstream implications for atherogenesis and microvascular pathology. In the reproductive system, a dedicated review highlighted arsenic-associated oxidative stress as a key contributor to male and female reproductive disorders, affecting hormone balance, gametogenesis, and gonadal function [183]. Complementing this, work synthesizing male fertility outcomes under arsenic exposure emphasizes ROS-mediated injury to reproductive tissues and sperm endpoints, reinforcing that reproductive impairment can be framed as part of the broader oxinflammation spectrum [184].

### 6.3. Cadmium

Cd is classified as a critically toxic heavy metal for biological systems [185]. It occurs naturally in the Earth’s crust, but anthropogenic activities increase its release into the environment [21]. Industrially, it is used in the manufacture of Ni-Cd batteries, pigments, and polymer stabilizers [186]. The main routes of exposure to the metal are inhalation and ingestion of contaminated food and water [21,187]. With a half-life of 20 to 30 years [188], Cd is water-soluble and highly toxic even at low concentrations [186]. Cd can be found in oxidation states 0, +1, and +2 [189], but the most stable and common oxidation state is +2 (Cd^2+^) [21], which is found as chloride, nitrate, or sulfate [190].

The oxinflammation process mediated by Cd causes damage to several systems. In the lungs, inhaled Cd is transported by ZIP-8 and DMT1, facilitating its distribution throughout the body [190]. Its distribution to other organs occurs through the bloodstream, bound to proteins, and through the erythrocyte membrane, with the liver and, mainly, the kidneys being the most critical sites of bioaccumulation of this metal. Its excretion rate in urine and feces is low, about 0.01–0.02% per day of the body load, which explains its prolonged biological half-life []. Hepatocytes are a primary target of this metal’s damage [55,187], with increased 8-Oxo-dG, MDA, and carbonyl protein content, indicating liver dysfunction. The kidneys accumulate a high concentration of Cd [55], and data suggest that a large proportion of the Cd retained in these organs is associated with MTs [191]. In the cardiovascular system, Cd promotes endothelial dysfunction and atherosclerosis, leading to cardiovascular complications such as hypertension, heart failure, and myocardial infarction [192]. In the nervous system, it can cross the blood–brain barrier [193] and cause symptoms such as headache, dizziness, and memory impairment [13]. In the reproductive system, its endocrine-disrupting action is noteworthy. In the female reproductive system, it promotes hormonal imbalance, affecting the menstrual cycle and generating adverse effects in offspring [194]. In the male reproductive system of mice, Cd exposure is dose- and time-dependent, leading to severe pathologies in the seminiferous tubules and testicular interstitium [195]. Epidemiological studies in humans indicate hormonal impairment, impaired spermatogenesis, and reduced sperm quality, factors that critically impact reproductive health [194].

### 6.4. Lead

Pb is a metal that humans and animals can be contaminated by inhaling Pb particles from smelting processes, recycling, and vehicle engine batteries, or by ingesting contaminated particles present in soil, water, and food [29]. When absorbed, 99% of Pb binds to red blood cells, while 1% remains in the plasma, where it is distributed mainly to bones and teeth [196]. Its most biologically relevant form is Pb^2+^, which can enter the cell through Ca^2+^ channels that mimic Ca^2+^ and through DMT1, which normally transports Fe^2+^ [23,197].

The hematopoietic system is one of the main targets of Pb toxicity, which critically interferes with heme biosynthesis by affecting enzymes such as δ-aminolevulinate synthase (δ-ALAS), δ-aminolevulinate dehydratase (δ-ALAD), and ferrochelatase [198]. Inhibition of δ-ALAD leads to the accumulation of δ-ALA, which is rapidly oxidized and generates radicals such as O_2_^•−^ and H_2_O_2_ [199]. In the liver, Pb-induced oxinflammation manifests as mitochondrial disruption. Pb^2+^ can alter complex III of the electron transport chain, resulting in electron leakage and ROS generation. In the neurological context, after replacing Ca ions, Pb^2+^ becomes competent to cross the blood–brain barrier, accumulating in glial cells [200]. In the liver, Pb-induced oxinflammation manifests as mitochondrial dysfunction [201]. In the reproductive system, particularly in the testes, Pb induces oxinflammation by inhibiting CAT and SOD via binding to -SH groups, leading to the generation of more ROS with subsequent testicular dysfunction and spermatogenesis associated with the inhibition of gene expression involved in the synthesis of steroid hormones in the testes [202]. In the cardiovascular system, Pb exposure induces hypertension [203]. In a study by Vaziri [204], the reduction in NO bioavailability due to Pb-induced oxidative stress is associated with a compensatory increase in endothelial NOS (eNOS) in the renal and cardiac parenchyma.

### 6.5. Mercury

Hg is an environmentally persistent and highly toxic heavy metal that occurs in multiple chemical forms, each with distinct toxicokinetic and toxicodynamic properties [205]. Elemental mercury (Hg^0^) is the only liquid metal at room temperature and exhibits a high vapor pressure, favoring its volatilization [206]. The oxidation of Hg^0^ gives rise to inorganic species, predominantly divalent mercury (Hg^2+^), capable of forming salts when associated with O, Cl, or S. Hg^2+^, the main cationic species of Hg in the environment, can also give rise to organic compounds by covalent bonding to groups such as methyl, ethyl, or phenyl, notably methylmercury (MeHg) [205,206]. The bioavailability, systemic distribution, and toxic potential of Hg depend directly on its chemical form and the route of exposure. Hg^0^ is predominantly absorbed by inhalation and rapidly distributed into the systemic circulation after pulmonary deposition, where it is oxidized to Hg^2+^ by enzymes such as CAT, thus sharing toxicity mechanisms with the inorganic forms of the metal [205,207]. Hg^2+^ has a lower capacity to cross biological barriers than MeHg. However, it can be efficiently internalized after forming conjugates with thiolated ligands [31,205,208]. Similarly, MeHg forms stable conjugates with cysteine and other thiols, which mimic neutral amino acids and are transported by specific systems, favoring their delivery to target tissues, especially the central nervous system [205,209].

In the central nervous system, MeHg-induced mitochondrial dysfunction sustains the continuous production of reactive species, compromising the homeostasis of neurons and glial cells. This pro-oxidant state acts as a signal for microglia activation and the induction of inflammatory pathways, contributing to neuroinflammatory and neurodegenerative processes [210]. Similarly, in the kidneys, Hg-induced redox imbalance precedes the activation of NF-κB-dependent inflammatory pathways and the NLRP3 inflammasome, with increased expression of pro-inflammatory cytokines such as TNF-α and IL-1β, culminating in tubular injury [211]. Similar mechanisms are also observed in the liver and cardiovascular system. In hepatocytes exposed to HgCl_2_, mitochondrial dysfunction, associated with excessive ROS production, precedes the activation of the NLRP3 inflammasome and the NF-κB pathway, characterizing inflammation as a secondary response to initial oxidative damage [212]. In the cardiovascular system, evidence indicates that Hg exposure compromises the mitochondrial bioenergetics of cardiomyocytes, favoring persistent oxidative stress and the activation of pro-inflammatory signaling [213]. In the female reproductive system, exposure to Hg reduces cell viability. It intensifies the production of reactive species in trophoblasts and endometrial cells, increasing cell death and DNA damage, and impairing critical processes such as adhesion, invasion, and trophoblast growth [214]. In the male reproductive system, exposure to HgCl_2_ increases the production of reactive species and inflammatory biomarkers, such as myeloperoxidase, evidencing the activation of inflammatory responses secondary to redox damage induced by the metal [215].

### 6.6. Nickel

Ni is a transition metal widely distributed in nature and used in various industrial applications, such as the manufacture of stainless steel, metal alloys, and batteries [40,216]. From a toxicokinetic point of view, Ni is predominantly absorbed in its divalent form (Ni^2+^) and transported in plasma bound to proteins such as albumin [217]. Soluble compounds, such as nickel chloride and nickel sulfate, are rapidly absorbed, while particulate or insoluble forms tend to persist in tissues [218].

At the systemic level, Ni-induced oxinflammation manifests in several organs. In the liver, mitochondrial dysfunction, chronic inflammation driven by exacerbated pro-inflammatory cytokine expression, and reprogramming of lipid metabolism are observed, with alterations in liver injury markers [219]. In the male reproductive system, increased reactive species, local inflammation, and impaired steroidogenesis lead to compromised spermatogenesis and reduced sperm quality [220,221]. In the female reproductive system, nickel exposure is associated with hormonal changes, ovarian inflammation, reduced antioxidant enzyme activity, increased markers of oxidative stress, and ovarian cell apoptosis, thereby compromising oocyte quality and ovarian homeostasis [222]. In the cardiovascular system, Ni is associated with endothelial dysfunction, inflammatory activation, and persistent oxidative stress, favoring tissue remodeling and functional alterations [36,223]. In the nervous system, exposure to Ni is associated with neuroinflammation, oxidative stress, mitochondrial damage, and behavioral changes, including cognitive deficits and neurodegeneration [224]. In the kidneys, exposure to Ni compounds is associated with tubular and glomerular dysfunction, elevated markers of oxidative stress, reduced antioxidant mechanisms, and activation of inflammatory responses [225]. Together, these effects reinforce the role of Ni as an inducer of an integrated oxinflammatory response, sustained by interactions between metabolism, epigenetics, and innate immunity.

## 7. Conclusions

In conclusion, this review reveals that heavy metal exposure induces a pro-oxidant state and initiates a cascade of events that characterizes the metal–cell interaction. Early disruption of redox homeostasis generates metabolites from oxidative damage, which act as central mediators in the activation and amplification of inflammatory responses. This sequence of events addresses the main question of this study, indicating that oxidative stress is the primary pathway of cellular damage, preceding the activation of inflammatory responses. The latter response emerges as a subsequent process, activating NF-kB and NLRP3 pathways and releasing pro-inflammatory cytokines, which, in turn, lead to ROS overproduction and amplify tissue damage. Therefore, both processes occur in an integrated and self-sustaining manner, characterizing a state of oxinflammation in which metabolites play a fundamental role in maintaining and propagating cellular imbalance. The metabolic reprogramming affects pathways of amino acid metabolism, lipid remodeling, energy production, and the generation of redox-, inflammatory-, and microbiota-derived metabolites. Metabolomic signatures stand out as tools for capturing the systemic response to toxin exposure, providing potential biomarkers of heavy metal-induced dysfunction. This perspective highlights metabolism as a hub linking oxidative stress to inflammation, reinforcing the relevance of metabolomics for advancing our understanding of metal-induced toxicity.

## Figures and Tables

**Table 1:** Representative metabolomic studies investigating the mechanisms of heavy metal-induced metabolic remodeling.

Heavy Metal	Sample (Murine Model/Organ)	Dose and Exposure Time	Metabolomics and Other Omics Approaches	Main Altered Metabolites	Main Changes in Metabolic Profiles and Other Aspects	Reference
Aluminum	Mouse; brain cortex	0.5 mg/m^3^ (whole-body inhalation, 6 h/day for 28 days) *	GC-MS-based untargeted metabolomics	Glutamate metabolism-related molecules (e.g., L-glutamic acid and pyroglutamic acid); acetyl-L-carnitine and riboflavin	Alterations in glutamate metabolism and neurotransmitter-related metabolites	[145]
	Mouse; astrocytes	125 μg/mL for 72 h in vitro *	LC-MS/MS-based untargeted metabolomics	Inflammatory responses (e.g., tyrosine, tryptophan, arginine, proline, and glycerophospholipids metabolic pathways) and oxidative stress (e.g., biosynthesis of valine, leucine and isoleucine and metabolism of tryptophan and phenylalanine)	Disturbances in amino acids, lipids, purine and pyrimidine metabolism associated with oxidative stress and inflammation	[146]
	Mouse; gut content	20 and 50 g/kg in the diet for 120 days	GC-MS-based untargeted metabolomics and microbiome integration	Amino acid metabolism (e.g., lysine, proline and putrescine), serotonin, and cholesterol	Changes in amino acids in the gut content, including cholesterol metabolism in the liver, and inflammatory pathways in the brain	[147]
	Rat; serum, gut content	1, 10, and 100 mg/kg by gavage for 28 days *	LC-MS/MS-based untargeted metabolomics and microbiome integration	Amino acid metabolism (e.g., D-proline, L-threonine, and L-phenylalanine); lipid metabolism (e.g., erucic acid, stearic acid, arachidonic acid)	Alterations in microbiota composition involved lipid and amino acid metabolism	[148]
Arsenic	Mouse; plasma, liver, kidney	3 mg/kg/day for 12 days	Direct infusion MS-based metabolomics (DIMS)	Energy metabolism (e.g., glucose, glyceraldehyde-3-phosphate, pyruvate, and citric acid); amino acid metabolism (e.g., arginine, tryptophan, cysteine, glutamic acid, and methionine); lipid metabolism (e.g., choline and phosphorylcholine)	Alterations in energy metabolism (glycolysis and TCA cycle), amino acid metabolism, purine metabolism and membrane phospholipid metabolism	[149]
	Rat; testis	1, 5, and 25 mg/L for 6 months	UPLC-MS-based metabolomics and proteomics integration	Amino acid metabolism (e.g., L-leucine and L-methionine); purine metabolism (e.g., hypoxanthine and inosine); L-acetylcarnitine	Alterations in metabolites associated with spermatogenesis and fertilization	[75]
	Rat; gut content	0.05, 0.25, 1.25, and 6.25 mg/L for 30 days	UPLC-MS-based untargeted metabolomics and microbiome integration	Lipid profile changes (e.g., lysoPC, lysoPE, PC and diacylglycerol); amino acid metabolism (e.g., L-arginine, glutathione and trimethyllysine)	Changes in glycerophospholipid metabolism, linoleic acid metabolism and amino acid biosynthesis	[150]
	Rat; liver	50 mg/kg for 90 days	LC-MS/MS-based untargeted metabolomics and ICP-MS-based ionomics integration	Porphyrin metabolism (e.g., coproporphyrin III and protoporphyrinogen); steroid hormone (e.g., progesterone, androsterone glucuronide and 7α,25-dihydroxycholesterol); taurine and hypotaurine metabolism (e.g., 3-sulfino-L-alanine and 5-L-glutamyl-taurine)	Disturbances in nicotinate and nicotinamide metabolism, one-carbon pool of folate, porphyrin metabolism, steroid hormone biosynthesis, and taurine/hypotaurine metabolism	[76]
Cadmium	Rat; liver	0.7, 2, and 6 mg/kg/day by gavage for 90 days	GC-MS-based untargeted metabolomics	Amino acid metabolism (e.g., L-aspartic acid, L-proline and L-lysine); energy metabolism (e.g., butanedioic acid and malic acid); lipid metabolism (e.g., linolenic acid)	Alterations in amino acids, fatty acid and energy metabolism, and oxidative damage in the liver	[151]
	Mouse; neural stem cells	1.5 μM for 24 h in vitro	UPLC-MS-based untargeted metabolomics and transcriptomics integration	Lipid metabolism (e.g., ethanolamine and phosphatidylethanolamine); amino acid metabolism (e.g., arginine and proline)	Changes in arginine and proline metabolism, particularly glycerophospholipid metabolism, leading to a perturbed membrane function and signal transduction	[152]
	Mouse; spermatogonia	20 μM for 24 h in vitro	LC-MS-based untargeted metabolomics and transcriptomics integration	Amino acid metabolism (e.g., histidine, isoleucine, aspartic acid, proline and tyrosine)	Changes in amino acid metabolism and metabolic pathways linked to ER stress and apoptosis	[153]
	Mouse; liver, gut content	100 nM in drinking water for 12 weeks	LC-MS-based untargeted metabolomics and metagenomics integration	Bile acid metabolism (e.g., deoxycholic acid 3-glucuronide and cholic acid glucuronide); lipid metabolism (e.g., phosphatidylcholine, lysoPC, phosphatidylglycerols and dicarboxylic acids)	Disruption of gut microbiota, bile acid metabolism and lipid metabolic pathways, driving liver injury	[77]
Lead	Mouse; gut content	10 ppm in drinking water for 13 weeks	GC-MS-based untargeted metabolomics and microbiome integration	Vitamin E (α-tocopherol and γ-tocopherol); bile acid metabolism (e.g., cholic acid, ursodeoxycholic acid and deoxycholic acid); energy metabolism (e.g., glycerol-3-phosphate)	Disruptions in energy metabolism, vitamin E, bile acids, and nitrogen metabolism, impairments in the defense/detoxification mechanism and in oxidative stress pathways	[154]
	Mouse; serum, gut content	100 mg/L in drinking water for 12 weeks	LC-MS/MS-based untargeted metabolomics and microbiome integration	Bile acid metabolism (e.g., lithocholic acid, taurohyodeoxycholic acid and taurochenodeoxycholic)	Alterations in bile acid metabolism and gut microbiota composition linked to neuroinflammation	[155]
	Mouse; liver, gut content	50 mg/kg for 4 weeks	LC-MS/MS-based metabolomics, transcriptomics, and microbiome integration	Lipid metabolism (e.g., carnitine); antioxidant metabolites (e.g., glutathione, 11-cis-retinol, all-trans-13,14-dihydroretinol and ferulic acid)	Changes in lipid, amino acid, and nucleotide metabolism and enhancing oxidative and inflammatory pathways and detoxification capacities	[156]
Mercury	Mouse; blood	0.2 mg/kg/day subcutaneous injection for 10 days	QTOF-MS untargeted metabolomics	Energy metabolism (e.g., glucose and lactic acid); lipid metabolism (e.g., choline, phosphocholine, L-carnitine and diacylglycerol); amino acid metabolism (e.g., valine, arginine, creatine and glutamine)	Changes in energy metabolism, amino acid metabolism, membrane phospholipid breakdown and oxidative stress-related metabolites	[157]
	Rat; gut content	0.4 μg/mL for 24 h	UPLC-QTOF-MS-based untargeted metabolomics	Amino acids/neurotransmitter metabolism (e.g., serine, GABA, glutamate, L-tyrosine, glycine and aspartic acid)	Changes in neurotransmitter metabolism and amino acids induce inflammatory and immunological responses	[158]
Nickel	Mouse; colon	450 mg/L in drinking water for 16 weeks	LC-MS/MS-based untargeted metabolomics and microbiome integration	Amino acid metabolism (e.g., valine, tryptophan, lysine and pyroglutamic acid); lipid metabolism (e.g., arachidonic acid and stearidonic acid)	Changes in amino acid metabolism and lipid metabolic pathways	[159]
	Rat; serum	0.015, 0.06, and 0.24 mg/mL for 9 weeks *	LC-MS-based untargeted metabolomics and metagenomics integration	Lipid metabolism (e.g., linoleic acid docosapentaenoic acid and phosphatidylcholine); progesterone; ascorbate and aldarate metabolism	Alterations in lipid metabolism, bile acid metabolism, amino acid metabolism, nucleotide metabolism, and carbohydrate and progesterone metabolic pathways	[160]
	Mouse; serum and gut content	20 mg/kg every other day for 15 days via gavage, intraperitoneal injection, or nasal instillation	GC-MS-based targeted SCFA metabolomics	Lipid metabolism (e.g., acetate, propionate, isovalerate, and butyrate)	Disturbances in short-chain fatty acids metabolism and inflammatory signaling pathways	[161]

* nanoparticles.

## Data Availability

No new data were created or analyzed in this study.

## Abbreviations

The following abbreviations are used in this manuscript:

4-HNE	4-hydroxynonenal
8-Oxo-dG	8-oxo-2′-deoxyguanosine
ASC	Apoptosis-associated granule-like protein containing a CARD domain
Casp-1	Caspase-1
CAT	Catalase
COX-2	Cyclooxygenase-2
DAMPs	Damage-associated molecular patterns
DMT1	Divalent metal transporter 1
eNOS	Endothelial NOS
ER	Endoplasmic reticulum
ERK	Extracellular signal-regulated kinase
ERO1	Endoplasmic reticulum oxidoreductin 1
ETC	Electron transport chain
FAD	Flavin adenine dinucleotide
GPx	Glutathione peroxidase
GR	Glutathione reductase
GSH	Reduced glutathione
GSSG	GSH/oxidized glutathione
GST	Glutathione S-transferase
H_2_O_2_	Hydrogen peroxide
ICAM-1	Intercellular adhesion molecule 1
IL-6	Interleukin-6
IL-8	Interleukin-8
IL-18	Interleukin-18
IL-1β	Interleukin-1 beta
IKK	IκB kinase complex
iNOS	Inducible nitric oxide synthase
IP_3_R	Inositol triphosphate receptor
IRAK	IL-1 receptor-associated kinases
IsoLGs	Isolevuglandins
IκBα	Inhibitor of NF-κB
JNK	c-Jun N-terminal kinase
Keap1	Kelch-like ECH-associated protein 1
MAPKs	Mitogen-Activated Protein Kinases
MDA	Malondialdehyde
MREs	Metal-responsive elements
MTF-1	Metal-responsive transcription factor-1
MTs	Metallothioneins
MyD88	Myeloid Differentiation Primary Response 88
NF-κB	Nuclear factor-κB
NLRP3	NOD-like receptor family pyrin domain-containing 3
NO	Nitric oxide
NOXs	NADPH oxidases
Nrf2	Nuclear erythroid factor 2-related factor 2
O_2_•^−^	Superoxide radical
PDI	Protein disulfide isomerase
PKC	Protein kinase C
PPARα	Peroxisome proliferator-activated receptor alpha
PRRs	Pattern recognition receptors
PUFAs	Polyunsaturated fatty acids
QH2/Q	Ubiquinol/ubiquinone ratio
RET	Reverse electron transport
RIRR	ROS-induced ROS release
RNS	Reactive nitrogen species
ROS	Reactive oxygen species
SIRT1	Sirtuin 1
SOD	Superoxide dismutase
TLRs	Toll-like receptors
TNF-α	Tumor necrosis factor alpha
TRPV	Transient receptor potential channels, vanilloid subtype
UPR	Unfolded protein response
UPR	Flavin adenine dinucleotide
VCAM-1	Vascular cell adhesion molecule 1
ΔΨm	Reverse electron transport
ΔΨm	Mitochondrial membrane potential
δ-ALAD	δ-aminolevulinate dehydratase
δ-ALAS	δ-aminolevulinate synthase
